# Facile Synthesis of Core/Shell-like NiCo_2_O_4_-Decorated MWCNTs and its Excellent Electrocatalytic Activity for Methanol Oxidation

**DOI:** 10.1038/srep20313

**Published:** 2016-02-01

**Authors:** Tae-Hoon Ko, Kesavan Devarayan, Min-Kang Seo, Hak-Yong Kim, Byoung-Suhk Kim

**Affiliations:** 1 Department of Organic Materials & Fiber Engineering, Chonbuk National University, 567 Baekje-daero, Deokjin-gu, Jeonju-si, Jeollabuk-do 54896, Republic of Korea; 2 Department of BIN Convergence Technology, Chonbuk National University, 567 Baekje-daero, Deokjin-gu, Jeonju-si, Jeollabuk-do 54896, Republic of Korea; 3 Korea Institute of Carbon Convergence Technology, Jeonju 54852, Republic of Korea; 4 Department of Basic Sciences, College of Fisheries Engineering, Tamil Nadu Fisheries University, Nagapattinam 611001, India

## Abstract

The design and development of an economic and highly active non-precious electrocatalyst for methanol electrooxidation is challenging due to expensiveness of the precursors as well as processes and non-ecofriendliness. In this study, a facile preparation of core-shell-like NiCo_2_O_4_ decorated MWCNTs based on a dry synthesis technique was proposed. The synthesized NiCo_2_O_4_/MWCNTs were characterized by infrared spectroscopy, scanning electron microscopy, transmission electron microscopy, X-ray diffraction, and selected area energy dispersive spectrum. The bimetal oxide nanoparticles with an average size of 6 ± 2 nm were homogeneously distributed onto the surface of the MWCNTs to form a core-shell-like nanostructure. The NiCo_2_O_4_/MWCNTs exhibited excellent electrocatalytic activity for the oxidation of methanol in an alkaline solution. The NiCo_2_O_4_/MWCNTs exhibited remarkably higher current density of 327 mA/cm^2^ and a lower onset potential of 0.128 V in 1.0 M KOH with as high as 5.0 M methanol. The impressive electrocatalytic activity of the NiCo_2_O_4_/MWCNTs is promising for development of direct methanol fuel cell based on non-Pt catalysts.

The ever growing demand for next-generation clean and high-efficiency energy has inspired considerable efforts in the development of advanced alternative energy conversion and storage devices with the features of low cost and more importantly, environmental friendliness. Among them direct alcohol fuel cells (DAFC) are attractive due to their high energy conversion efficiency. Methanol has higher energy density than hydrogen. Abundant inexpensive sources are available for production of methanol and more over the direct methanol fuel cell (DMFC) possess high energy conversion efficiency and stability^1^,^2^,^3^,^4^. Currently platinum (Pt)-based electrocatalysts exhibit higher energy conversion but non-preferable due to its high cost and catalytic poisoning which reduce the catalytic activity^5^. The successful implementation of the fuel cells mainly depends on the economy, activity, and the durability of the electrocatalysts. Therefore, it is important to design highly efficient as well as economical electrocatalysts for practical applications. The development of non-Pt based electrocatalysts is still challenging mainly due to lack of high conductivity and excellent catalytic activity and stability of the catalyst. Most of the preceding researches report non-Pt based electrocatalysts using transition metal oxides, for instance, NiO^6^,^7^, Co_3_O_4_^8^. It has been reported that a strategy to combine both NiO and Co_3_O_4_ resulted in the high electrocatalytic efficiency than their individual counterparts. For example, NiCo_2_O_4_ exhibits excellent electrochemical activity due to higher electronic conductivity than either NiO or Co_3_O_4_^9^,^10^.

Several synthetic strategies, such as conventional hydrothermal^11^,^12^,^13^,^14^,^15^, solvothermal^16^,^17^,^18^, electrochemical synthesis^19^,^20^ were for the preparation of Ni and Co-based bimetal oxide electrocatalysts with different morphologies. However, most of them involve in use of toxic chemicals such as NH_4_F, high temperatures and other non-ecofriendly solvents. In this study, we have developed an economical, environmental friendly, dry synthesis method for facile preparation of core-shell-like NiCo_2_O_4_ decorated multiwall carbon nanotubes (MWCNT). The synthesized electrocatalyst demonstrated higher catalytic activity for electrooxidation of methanol. To the best of our knowledge NiCo_2_O_4_/MWCNT has not been synthesized via such a simple grinding method followed by low temperature annealing. Further this is the first study to demonstrate the use of NiCo_2_O_4_/MWCNT as an electrocatalyst for direct methanol fuel cell.

## Experimental

### Materials

MWCNT (>95% in purity, 20 ~ 25 μm in length, <20 nm in diameter) was kindly provided by nanosolution Co., Korea. Cobalt (II) acetate tetra hydrate (CoAc, 99.0% assay, Sigma-Aldrich), and nickel acetate tetra hydrate (NiAc, 99.0% assay, Sigma-Aldrich) were utilized without any further modification as precursors. Methanol, potassium hydroxide were purchased from Junsei Co. Ltd. (Japan). Millipore water (Milli-Q system) was used for the preparation of solutions.

### Synthesis of Core/Shell-like NiCo_2_O_4_-Decorated MWCNTs

At first, 0.2 g of pure MWCNTs were treated with 3 M HNO_3_ and then refluxed for 48 h to remove metal impurities. After cooling to room temperature, the solution was diluted with 500 mL of deionized water and then vacuum-filtered through a filter paper with the pore size of 0.45 μm. The resultant purified MWCNTs were washed with deionized water until the pH became neutral and then dried in vacuum at 80 °C for 48 h.

For the preparation of core-shell-like NiCo_2_O_4_-decorated MWCNTs, 0.2 g of purified MWCNT was ground using a mortar and pestle for 10 min. Then, 0.05 g of each CoAc and NiAc were added into the ground-MWCNTs and ground well. The homogeneous mixture of MWNTs and CoAc and NiAc was obtained in 15 min. Finally, the mixture was calcinated at 300 °C for 4 h in air atmosphere. The composition of the CoAc and NiAc were varied at a ratio of Ni:Co = 0.2 : 0.8, 0.4 : 0.6, 0.6 : 0.4, 0.2 : 0.8, 1.0 : 1.0 with fixing the total quantity as 0.1 g. The samples before calcination and after calcination were indicated by NiAc-CoAc/MWCNT and NiCo_2_O_4_/MWCNT, respectively.

### Characterization

The morphologies of all the samples were observed under a JEOL JSM-5900 scanning electron microscopy (SEM) after sputtering the samples with platinum for 120 s. Energy dispersive X-ray measurements were conducted using the EDAX system attached to the same microscope. A field-emission scanning electron microscope (FE-SEM) was also used to observe the morphologies after sputtering the samples with osmium for 7 s. For TEM, the sample was prepared by dispersing in ethanol by sonication at a concentration of 0.1 mg/mL. 1 mL of sample was dropped on the Cu TEM grid and analyzed. The FT-IR of spectra of the samples was recorded using a Perkin Elmer instrument. X-ray diffraction (XRD) patterns were recorded on a Rikaku X-ray diffractometer (Cu Kα radiation). The surface of the samples was analyzed by X-ray photoelectron spectroscopy (XPS, ESCALAB250, Al Kα radiation). VersaSTAT4 (USA) electrochemical analyzer and a conventional three-electrode electrochemical cell were utilized to investigate the electrochemical measurements. Pt wire and Ag/AgCl (saturated KC) were used as the counter and reference electrodes, respectively. The glassy carbon electrode (GCE, 3 mm diameter; area 0.07 cm^2^) was used as the working electrode. The MWNT/NiCo_2_O_4_ powder (2 mg) was dispersed in 2-propanol (400 μL) and sonicated for 5 min. And then the mixture was added nafion solution (20 μL) and sonicated for 5 min. Then the solution was dropped on the surface of the GCE and dried at 60 °C for 15 min. Cyclic voltammetry (CV) and chronoamperometry (CA) measurements were performed to study the activity and stability of methanol oxidation reaction. The test solutions used in this study were 1.0 M KOH solution with and without addition of various methanol concentrations. All the experiments were performed at 298 ± 2 K. The electrochemical impedance spectroscopy (EIS) was recorded in the frequency range of 10 mHz to 100 kHz with a potential amplitude of 10 mV.

## Results and Discussion

The chemical conversion of NiAc-CoAc/MWCNT into NiCo_2_O_4_/MWCNT was proved by means of FT-IR spectral changes before and after calcination (Fig. S1). The NiAc-CoAc/MWCNT exhibited characteristic peaks at 3128, 2885, 1522, and 1487 cm^−1^ corresponding to asymmetric stretching of C-H, symmetric stretching of C-H, O-C=O of acetate, and C-H deformation. Disappearance of these unique peaks in the FT-IR spectrum of NiCo_2_O_4_/MWCNT after calcination indicated the successful synthesis.

**Figure 1:**
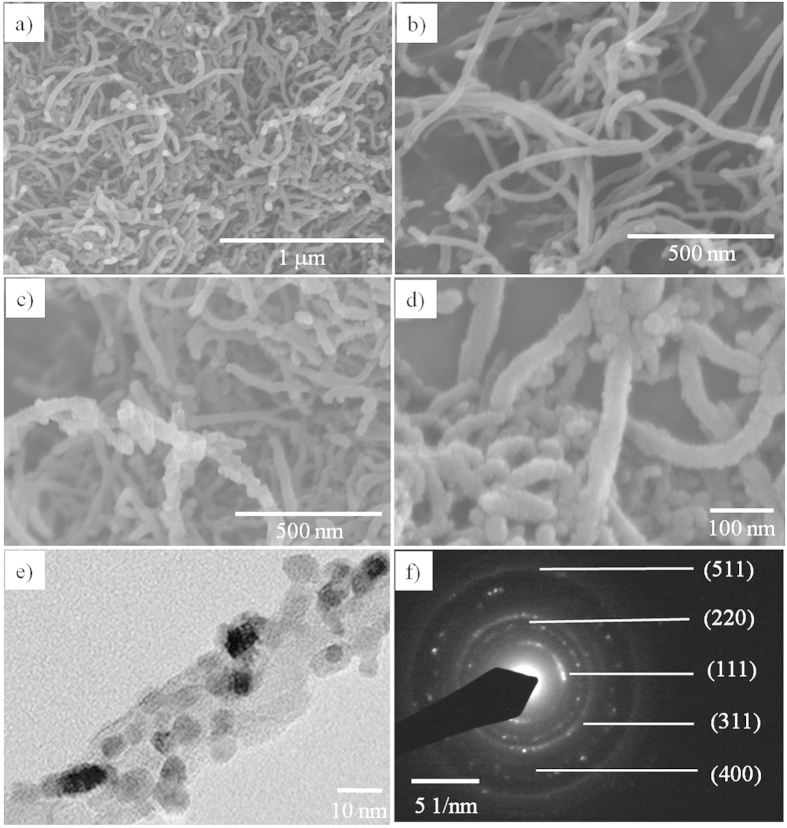
FE-SEM images of pristine MWCNT (**a,b**), NiCo_2_O_4_/MWCNT (**c,d**). HR-TEM image (**e**) and SAED pattern (**f**) of NiCo_2_O_4_/MWCNT.

The morphologies of the synthesized NiCo_2_O_4_/MWCNT were investigated by using FE-SEM and TEM measurements. Figure 1a,b showed the smooth surface morphologies of pristine MWCNT. On the other hand, the NiCo_2_O_4_/MWCNT exhibited a rough surface, suggesting the successful decoration of the MWCNTs with bimetal oxide nanoparticles (Fig. 1c–e). It was estimated that the thickness of the MWCNT increased from ~20 nm to 48 nm on average after decoration with NiCo_2_O_4_ nanoparticles. A close examination of the HR-TEM image of the NiCo_2_O_4_/MWCNT showed successful deposition of quasi-spherical NiCo_2_O_4_ nanoparticles on the MWCNTs, which is similar to the core (MWCNT)/shell (NiCo_2_O_4_) structure. The average size of the nanoparticles over the surface of MWCNTs was 6 ± 2 nm. Further, the SAED pattern (Fig. 1f) exhibited well-defined rings of (511), (220), (111), (311), and (400) suggesting the polycrystalline nature of the synthesized NiCo_2_O_4_/MWCNT (JCPDS no. 73–1702).

**Figure 2:**
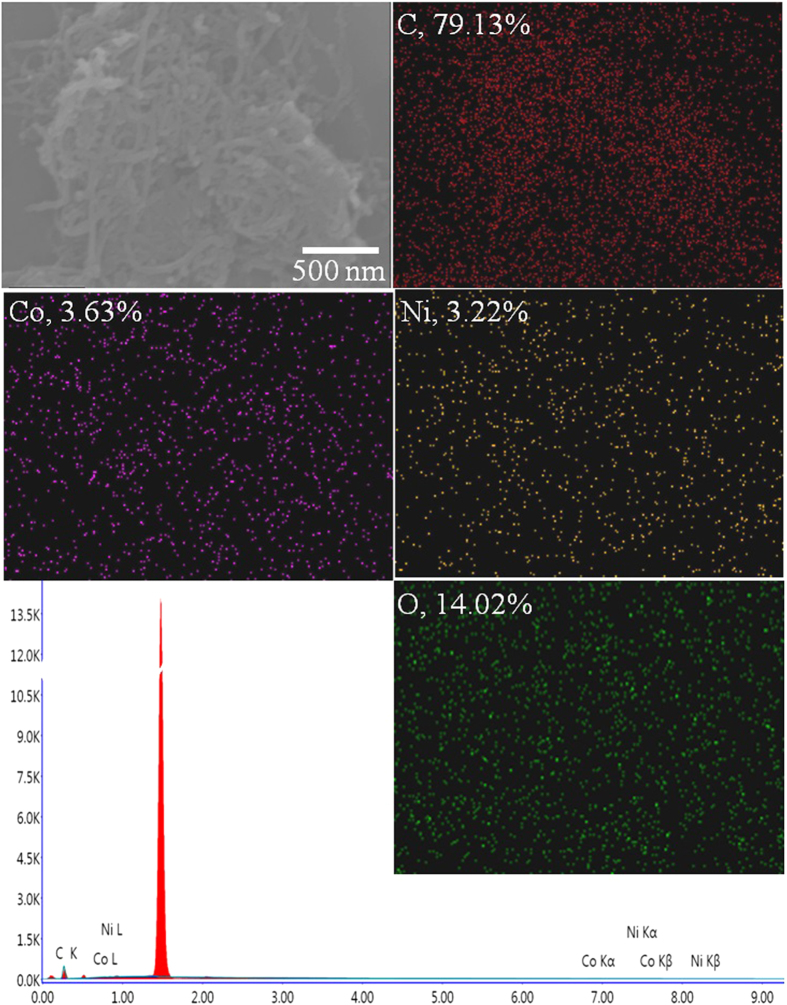
FE-SEM/EDS spectrum and mapping of NiCo_2_O_4_/MWCNT.

In order to determine the weight percentage (wt%) of Ni and Co in NiCo_2_O_4_/MWCNT and to inspect their distribution, FE-SEM/EDS and their corresponding elemental mapping images were taken for NiCo_2_O_4_/MWCNT (Fig. 2). The wt% of the elements were C, 79.13%; O, 14.02%; Ni, 3.22%; Co, 3.63%. The homogeneous distribution of Ni and Co in NiCo_2_O_4_/MWCNT indicated that the proposed synthesize method is effective and successful.

**Figure 3:**
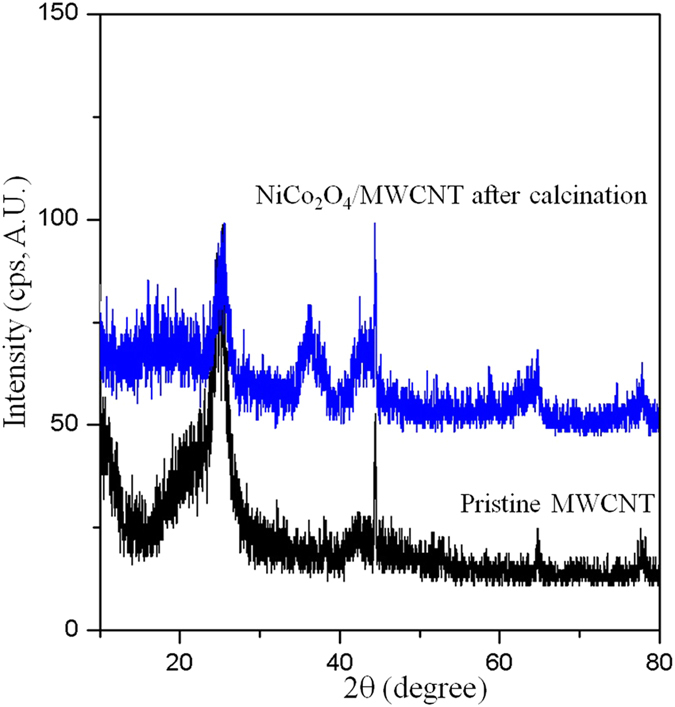
X-ray diffraction patterns of pristine MWCNT and NiCo_2_O_4_/MWCNT after calcination at 300 °C.

Figure 3 shows the XRD patterns of pristine MWCNT and NiCo_2_O_4_/MWCNT. In the case of pristine MWCNTs, the diffraction peaks were observed at 26.5^o^, 42.4^o^, and 64.7^o^, corresponding to the (002), (100), and (004), respectively, which is attributed to the hexagonal graphite structures of CNTs. On the other hand, the XRD profile of NiCo_2_O_4_/MWCNT exhibited peaks corresponding to the (111), (220), (311), (400), (422), (511), and (440) reflections of the spinel NiCo_2_O_4_ crystalline structure (JCPDS no. 73–1702).

**Figure 4:**
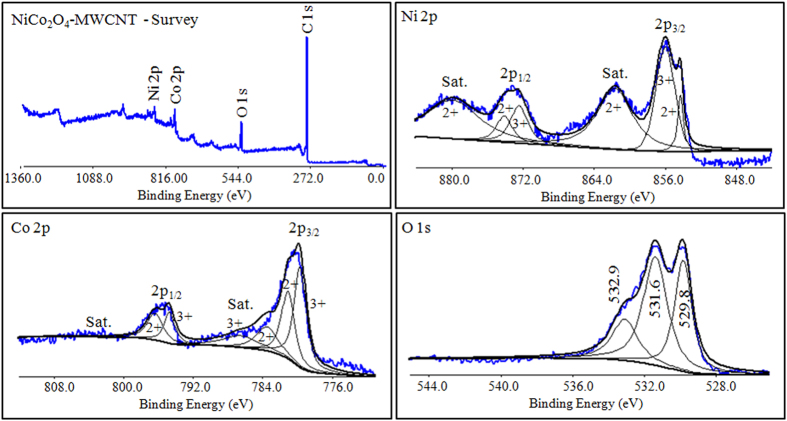
XPS spectrum of NiCo_2_O_4_/MWCNT: Survey spectrum (top-left), Ni 2p (top-right), Co 2p (bottom-left), O 1 s (bottom-right).

In order to understand the chemical composition and the oxidation states of the metals in NiCo_2_O_4_/MWCNTs, the samples were subjected to XPS analyses and the results are shown in Fig. 4. The survey spectrum indicated the presence of elements Ni, Co, O, and C as well as absence of any impurities. The Ni 2p spectrum best fitted with two spin-orbit doublets corresponding to the oxidation states of 2+ and 3+ of nickel along with two shake-up satellites (indicated as ‘Sat.) at the high binding energy side of the Ni 2p_3/2_ and 2p_1/2_ edge. Similarly, the spectrum of Co 2p also best fitted with two spin-orbit doublets that are corresponding to Co^2+^ and Co^3+^ and two shake-up satellites. Thus the results indicated that the NiCo_2_O_4_/MWCNT contains Ni^2+^/Ni^3+^ and Co^2+^/Co^3+^. The XPS results are in agreement with preceding literature for synthesis of NiCo_2_O_4_^21^,^22^,^23^,^24^. Further the O 1 s spectrum revealed the presence of metal oxygen bonds by exhibiting a peak at 529.8 eV. The peak at 532.9 eV is ascribed to the chemisorbed oxygen. The peaks at 529.8 and 531.6 eV are due to O^2−^ which indicates the formation of spinel NiCo_2_O_4_^23^,^24^.

**Figure 5:**
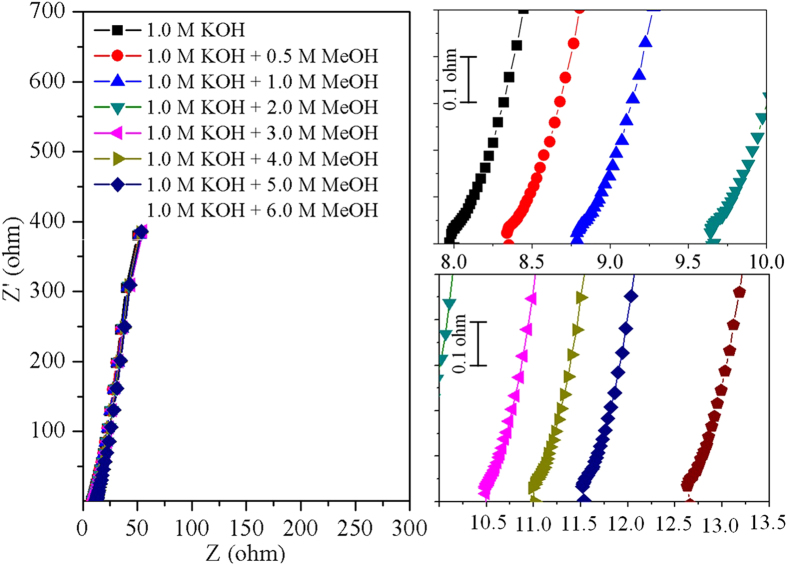
Impedance response of NiCo_2_O_4_/MWCNT in 1.0 M KOH, and 1.0 M KOH with 0.5 M, 1.0 M, 2.0 M, 3.0 M, 4.0 M, 5.0 M, and 6.0 M MeOH.

In general, the electrochemical performance of any material depends on the rate of electron transfer or charge transfer. The electron transfer kinetics of NiCo_2_O_4_/MWCNT was studied by the electrochemical impedance spectroscopy (EIS) in 1.0 M KOH with and without different concentrations of methanol (Fig. 5). The EIS curves exhibited a semicircle and a straight line. The semicircle is the characteristic behavior for charge transfer resistance of the material and the straight line is due to the reduction-oxidation behavior of nickel oxide and cobalt oxide^25^. It is noteworthy that the internal resistance of the material increased slightly in proportion to the increasing concentration of the methanol. Though the change in resistance between each solution is too small, it could be attributed to the effect of adsorption of the reactant and intermediate molecules on the catalyst.

**Figure 6:**
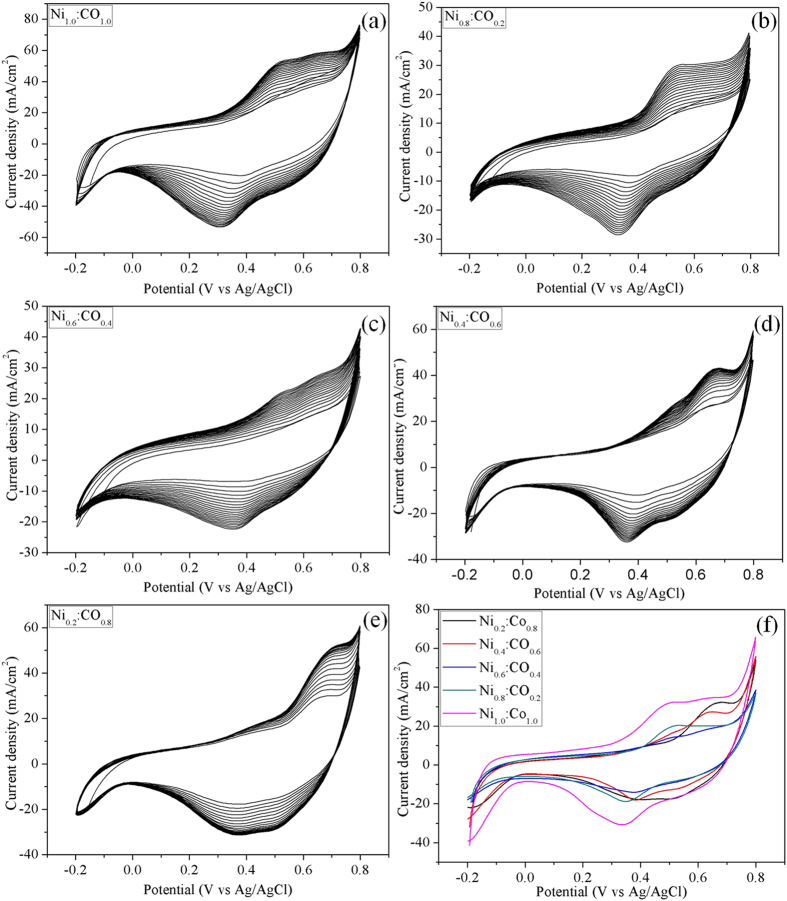
Cyclic voltammograms (first 20 cycles, (**a–e**) in presence of 1.0 M KOH at 100 mV/s for NiCo_2_O_4_/MWCNT with different Ni:Co ratios (Ni:Co = 0.2 : 0.8, 0.4 : 0.6, 0.6 : 0.4, 0.2 : 0.8, 1.0 : 1.0). (**f**) shows the cyclic voltammograms after 20 cycles at 50  mV/s.

It is noteworthy that surface activation of the NiCo_2_O_4_/MWCNT is important for better electrochemical activity^26^. Further, the composition ratio of the Ni and Co also influences the electrocatalytic activity. Therefore, the working electrode prepared with NiCo_2_O_4_/MWCNT having different Ni:Co ratio (0.2 : 0.8, 0.4 : 0.6, 0.6 : 0.4, 0.2 : 0.8, 1.0 : 1.0) were subjected to a pre-activation of 20 cycles in cyclic voltammetry at a scan rate of 100 mV/s (Fig. 6a–e). It is observed that all the CV curves consist of a pair of well-defined redox peaks. These peaks were attributed to the oxidation and reduction of Co(II) and Ni(III). It should be noted that the redox potentials of cobalt oxide and nickel oxide are close to each other. Therefore, the redox peaks of cobalt oxide and nickel oxide are not clearly distinguishable in the CV curve of NiCo_2_O_4_/MWCNTs (Fig. 6a–e). In the present study, NiCo_2_O_4_/MWCNT with Ni:Co=1.0 : 1.0 was a best performing material attributed to higher current density (32.72 mA/cm^2^) and lower oxidation peak potential (0.49 V) (Fig. 6f).

**Figure 7:**
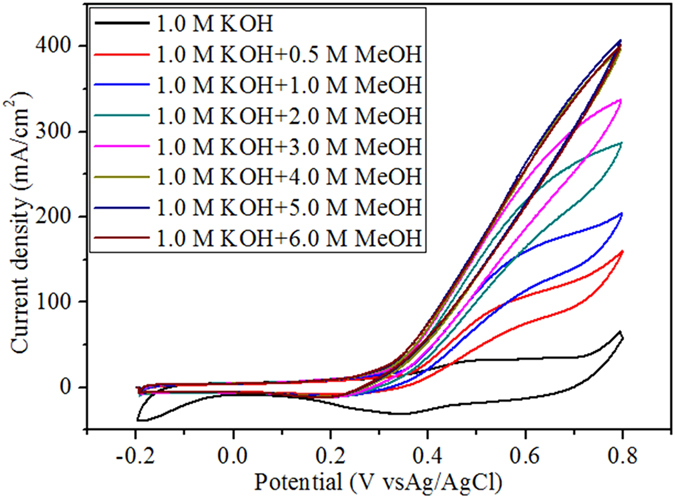
Electrocatalytic oxidation of methanol in 1.0 M KOH by NiCo_2_O_4_/MWCNT at 50 mV/s.

Figure 7 shows the electrochemical oxidation of MeOH by NiCo_2_O_4_/MWCNT with Ni:Co=1.0 : 1.0 in 1.0 M KOH at a scan rate of 50 mV/s. It is noteworthy that the addition of even 0.5 M MeOH increased the anodic peak current density to ~3 times (97.53 mA/cm^2^) with a peak potential of 0.553 V. It should be noted that the heterogeneous catalysis reaction involves in adsorption of the reactant on the catalyst followed by the formation of intermediate species and then products. Thus, the anodic peak observed in the curve of forward sweep was corresponding to the oxidation of the MeOH, whereas, the peak observed in the curve of reverse sweep was attributed to the oxidation of adsorbed intermediate species produced in the forward sweep.

The mechanism for electro-oxidation of methanol by NiCo_2_O_4_/MWCNT is supposed to be similar to nickel oxide and cobalt oxide catalysts as given below (Eq. 1–3)^27^,^28^,^29^,^30^.


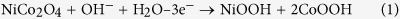



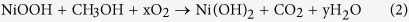



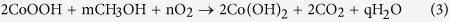


The concentration of alcohol is one of the important parameter to determine the performance of the fuel cell. Generally, DAFCs working with highly concentrated alcohol solution is preferable, since it could dramatically decrease the size of the fuel cell and simultaneously increase the power density. Further, it is impossible to use absolute methanol as the anodic reaction (Eq. 1) because it requires water. Unfortunately, most of the preceding DMAC studies involving NiCo_2_O_4_ based catalysts have been reported to use lower concentrations of MeOH such as 0.5 M to 3.0 M MeOH, which do not show any performance beyond this concentration threshold. Interestingly, in the present study, NiCo_2_O_4_/MWCNT exhibited electrocatalytic performance up to 5.0 M MeOH with a high current density of 327 mA/cm^2^ and peak potential of 0.675 V (Table 1). For 6.0 M MeOH, the current density slightly decreases, which indicate that the concentration threshold in this study is 5.0 M MeOH. To the best of our knowledge, this is the highest alcohol concentration ever to be reported, especially for NiCo_2_O_4_ -based catalysts for methanol electro-oxidation.

**Figure 8:**
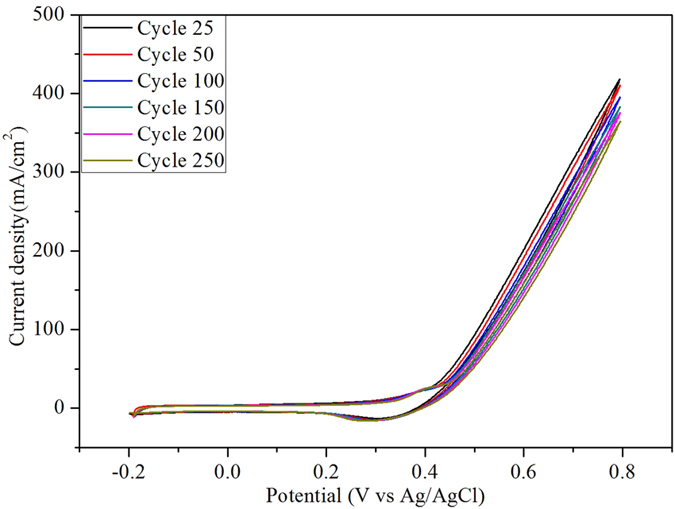
Cyclic voltammograms of NiCo_2_O_4_/MWCNT after 50, 100, 150, and 200 cycles at 100 mV/s in 1.0 M KOH with 6.0 M MeOH.

Onset potential is the other important parameter to demonstrate the electrocatalytic activity of the catalyst since it is the indicative measure for over potential. Pt-based catalysts perform better than non-precious electrocatalysts due to their smaller onset potential. In the present study, the onset potentials range from 0.128 to 0.262 V. The small organic molecules that were adsorbed on the catalyst and the molecules which are not fully oxidized are ascribed for the slight increase in the onset potentials. However, the onset potential reported in this study is one of the lowest values reported in the literature so far. Further to study the stability of the electrocatalytic activity, the NiCo_2_O_4_/MWCNT was subjected to longer CV cycles in 1.0 M KOH with 5.0 M MeOH. Figure 8 displays the good stability of NiCo_2_O_4_/MWCNT even after 250 cycles with retaining current density over 76%. Table 2 shows the anodic peak potentials of different Ni and Co based electrocatalysts for methanol oxidation. It is noteworthy that the high performance of the synthesized core-shell-like NiCo_2_O_4_/MWCNT was due to the fine size of the metal oxide nanoparticles and their homogeneous distribution on MWCNTs.

## Conclusions

In this work, we report a new and easy synthetic route to prepare core-shell-like NiCo_2_O_4_/MWNTs via a dry synthesis method. The efficient electrocatalytic oxidation of methanol on NiCo_2_O_4_/MWNTs was studied by cyclic voltammetry in 1.0 M KOH in the presence and absence of methanol. The NiCo_2_O_4_/MWNTs exhibited an impressively high electrocatalytic activity for methanol oxidation as the corresponding current increased with increasing the methanol concentration in the alkaline medium. This electrocatalyst is active for up to 5.0 to 6.0 M methanol. Remarkably this catalyst revealed a small onset potential lesser than 0.128 V vs Ag/AgCl, which is one of the superior value among the reported non-precious electrocatalyst. Overall, this work opens up opportunities for facile preparation of non-precious metal electrocatalysts via an economical, simple, and eco-friendly synthetic route with high catalytic activity.

## Additional Information

**How to cite this article**: Ko, T.-H. *et al.* Facile Synthesis of Core/Shell-like NiCo_2_O_4_-Decorated MWCNTs and its Excellent Electrocatalytic Activity for Methanol Oxidation. *Sci. Rep.* **6**, 20313; doi: 10.1038/srep20313 (2016).

## Supplementary Material

Supplementary Information

## Figures and Tables

**Table 1:** Influence of methanol concentration on electrooxidation parameters.

Parameters		Methanol concentration (M)						
	0	0.5	1	2	3	4	5	6
Peak potential (V)	0.49	0.553	0.58	0.657	0.664	0.674	0.675	0.678
Current density (mA/cm^2^)	32.72	97.53	153.5	247.9	283.3	324.2	327.3	322.5
Onset potential (V)	—	0.262	0.16	0.128	0.133	0.157	0.156	0.156

**Table 2:** Comparison of the electrocatalytic parameters for electrooxidation of methanol using different electrodes.

Electrocatalyst	Anodic peak potential (V)	Peak current density (mA/cm^2^)	Methanol Concentration Threshold (M)	Reference
NiCo_2_O_4_-RGO	0.6	~0.05	0.5	17
NiCo_2_O_4_ spinel nanoparticles	0.6	93	0.5	11
NiCo_2_O_4_/Ni foam – Nanosheet	0.6	111	0.5	15
NiCo_2_O_4_/Ni foam – Nanocloth	0.6	134	0.5	15
Porous NiCo_2_O_4_	0.6	98	0.5	14
Ni-Co alloy	0.6	2.3	0.5	31
NiCo_2_O_4_	0.65	0.5	0.05	32
NiCo_2_O_4_/MWCNT	0.55–0.68	97.3–327.3	5	Present study
