# Supplementary material for: Facile Synthesis of Core/Shell-like NiCo2O4-Decorated MWCNTs and its Excellent Electrocatalytic Activity for Methanol Oxidation
Source: Sci Rep. 2016 Feb 1;6:20313. doi: 10.1038/srep20313 (PMC4734329; doi:10.1038/srep20313)
Supplement: Supplementary Information [file srep20313-s1.pdf]

SUPPLEMENTARY INFORMATION

Facile Synthesis of Core/Shell-like $NiCo_2O_4$-Decorated MWCNTs and its Excellent Electrocatalytic Activity for Methanol Oxidation

Tae-Hoon Ko[a#], Kesavan Devarayan[b,d#], Min-Kang Seo[c], Hak-Yong Kim[a,b], Byoung-Suhk Kim[a,b*]

[a] Department of Organic Materials & Fiber Engineering, [b] Department of BIN Convergence Technology, Chonbuk National University, 567 Baekje-daero, Deokjin-gu, Jeonju-si, Jeollabuk-do 54896, Republic of Korea

[c] Korea Institute of Carbon Convergence Technology, Jeonju 54852, Republic of Korea

[d] Department of Basic Sciences, College of Fisheries Engineering, Tamil Nadu Fisheries University, Nagapattinam 611001, India

*Corresponding Author: Byoung-Suhk Kim; E-mail: kbsuhk@jbnu.ac.kr

[#] Authors are equally contributed.

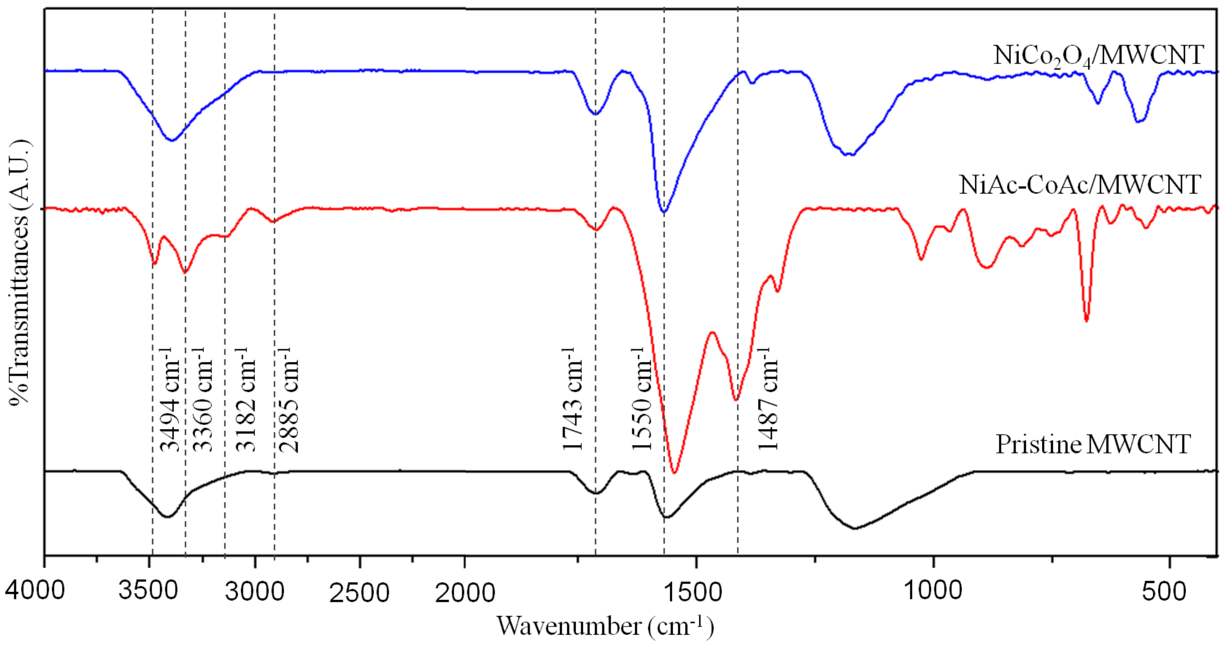

Figure S1. FT-IR spectra of pristine MWCNT, NiAc-CoAc/MWCNT, and $NiCo_2O_4$/MWCNT.
